# Células troncales cancerígenas en carcinoma oral de células escamosas. Revisión de la literatura

**DOI:** 10.21142/2523-2754-1002-2022-106

**Published:** 2022-06-27

**Authors:** Mariana Paulina Rodríguez-Vargas, Hugo Alvarado-Garnica, Luis David Gutiérrez-Verdín, Francisco Germán Villanueva-Sánchez, René García-Contreras

**Affiliations:** 1 Laboratorio de Investigación Interdisciplinaria (LII), Área de Patología Oral y Maxilofacial. Escuela Nacional de Estudios Superiores (ENES) Unidad León, Universidad Nacional Autónoma de México. León, Guanajuato, México. rodriguezvargasp@gmail.com, fgvillanuevas@enes.unam.mx Universidad Nacional Autónoma de México Laboratorio de Investigación Interdisciplinaria (LII), Área de Patología Oral y Maxilofacial Escuela Nacional de Estudios Superiores (ENES) Unidad León Universidad Nacional Autónoma de México León Guanajuato Mexico rodriguezvargasp@gmail.com fgvillanuevas@enes.unam.mx; 2 Laboratorio de Investigación Interdisciplinaria, Área de Nanoestructuras y Biomateriales. Escuela Nacional de Estudios Superiores (ENES) Unidad León, Universidad Nacional Autónoma de México. León, Guanajuato, México. hugotsuenum@gmail.com, rgarciac@enes.unam.mx Universidad Nacional Autónoma de México Laboratorio de Investigación Interdisciplinaria, Área de Nanoestructuras y Biomateriales Escuela Nacional de Estudios Superiores (ENES) Unidad León Universidad Nacional Autónoma de México León Guanajuato Mexico hugotsuenum@gmail.com rgarciac@enes.unam.mx; 3 División de Ciencias e Ingenierías Campus León, Universidad de Guanajuato. León, Guanajuato, México. luisdagnr94@gmail.com Universidad de Guanajuato División de Ciencias e Ingenierías Campus León Universidad de Guanajuato León Guanajuato Mexico luisdagnr94@gmail.com

**Keywords:** carcinoma de células escamosas de cabeza y cuello, células madre neoplásicas, transición epitelio-mesenquima, inmunohistoquímica, terapia molecular dirigida, squamous cell carcinoma of head and neck, neoplastic stem cells, epithelial-mesenchymal transition, immunohistochemistry, molecular targeted therapy

## Abstract

**Objetivo:**

Describir, mediante una revisión de la literatura, el carcinoma oral de células escamosas, la presencia de células troncales cancerígenas, su asociación con el curso de la enfermedad y las aplicaciones terapéuticas.

**Método:**

Se realizó una búsqueda en la base de datos PubMed introduciendo el siguiente algoritmo: *(((neoplastic stem cells [ MeSH Terms ]) OR (Cancer stem cells [Text Word ])) AND (Squamous Cell Carcinoma of Head and Neck [MeSH Terms ])) AND (Oral squamous cell carcinoma [Text Word ])*, para hallar artículos en idioma inglés publicados entre los años 2012 y 2022. Se utilizó el diagrama PRISMA para la identificación y elección de los artículos.

**Resultados:**

Se obtuvo un resultado de 49 artículos, de los cuales se eligieron 27 según su título y resumen, y su asociación con el tema. Se incluyeron 8 artículos adicionales sugeridos por su relación con la información previamente buscada, con lo que sumaron un total de 35 artículos evaluados. Se encontró que las células tumorales en el carcinoma oral de células escamosas son heterogéneas, pues incluyen células troncales cancerígenas, las cuales poseen características de células troncales, así como de células neoplásicas. Se las ha asociado con la progresión de la enfermedad, la recurrencia y la metástasis, y se ha considerado que son un mecanismo clave en el fallo en la terapia.

**Conclusiones:**

Se han identificado células troncales en carcinomas orales de células escamosas, las cuales han mostrado la expresión de marcadores, los cuales aportaron en su identificación y se han implicado en el comportamiento de las células cancerosas. Se han propuesto y desarrollado nuevas medidas terapéuticas dirigidas a la eliminación de las células troncales cancerígenas.

## Abstract (en)

**Objective:**

To perform a literature review on oral squamous cell carcinoma, the presence of cancer stem cells; their association with the course of the disease and therapeutic applications.

**Methods:**

: A search was performed in the PubMed database by entering the following algorithm: ((((neoplastic stem cells [MeSH Terms ]) OR (Cancer stem cells [Text Word ])) AND (Squamous Cell Carcinoma of Head and Neck [MeSH Terms])) AND (Oral squamous cell carcinoma [Text Word ]), to find articles in english published between 2012 and 2022. The PRISMA diagram was used to identify and select the articles.

**Results:**

A result of 49 articles was obtained; of which 27 were chosen according to the title and abstract in their association with the topic. In addition, 8 additional articles suggested by their relationship with the information previously searched were included. In total, 35 articles were evaluated. There has been found that tumoral cells in squamous oral carcinoma are heterogeneous since they include cancer stem cells wich possess characteristics of stem and neoplasic cells; which possess characteristics of stem cells as well as neoplastic cells; they have been associated with disease progression, recurrence, and metastasis and have been considered to be a key mechanism of therapy failure.

**Conclusions:**

The expression of stem cell markers in oral squamous cell carcinomas has been demonstrated and has contributed to their identification in oral squamous cell carcinomas and has been implicated in the behavior of cancer cells. New therapeutic measures aimed at eliminating cancer stem cells have been proposed and developed.

## INTRODUCCIÓN

El carcinoma oral de células escamosas (COCE), junto con aquellos de aparición en faringe y laringe, forma parte de los carcinomas de células escamosas de cabeza y cuello, que se desarrollan a partir del epitelio mucoso de dichas estructuras. Se trata de la neoplasia maligna más común de esta región anatómica ^1^. Se reporta que las neoplasias localizadas en la orofaringe se asocian, en mayor proporción, con una infección previa por cepas oncogénicas del virus del papiloma humano, principalmente VPH-16 y, en menor medida, VPH-18. Por su parte, aquellos que tienen origen en la cavidad oral y la laringe se relacionan en mayor medida con el tabaquismo ^2^.

El carcinoma de células escamosas de cabeza y cuello es la sexta neoplasia maligna más común alrededor del mundo ^3^. La tasa de supervivencia a 5 años continúa en torno al 40 y 60% ^4^. La progresión de este cáncer, la recurrencia, la metástasis y la resistencia a la terapia se explican al asociarlas con las células malignas, que presentan amplia heterogeneidad. Dentro de esta variedad de células tumorales existen células madre cancerígenas que impulsan el desarrollo del carcinoma ^5^^,^^6^. 

Las células madre cancerígenas son células tumorales que tienen características tanto de células troncales como de células neoplásicas. Debido a su presencia y su capacidad para esquivar el tratamiento, pueden ser un mecanismo clave en el fallo en la terapia ^7^. El carcinoma oral de células escamosas puede surgir de novo; sin embargo, muchos son precedidos por la presencia de cambios clínicos en la apariencia de la mucosa oral, que se pueden definir como trastornos orales potencialmente malignos. Por lo tanto, se requieren mejores marcadores como predictores de una transformación maligna ^8^.

El propósito de esta investigación fue ejecutar una revisión de la información reportada recientemente acerca del carcinoma oral de células escamosas, con enfoque en la evaluación del papel de las células troncales neoplásicas en el proceso de carcinogénesis, su identificación molecular y sus potenciales opciones terapéuticas.

Este trabajo pretende describir, de manera general, la participación crucial de las células troncales neoplásicas en el desarrollo y la invasión tumoral, la resistencia al tratamiento y los resultados en el manejo de la enfermedad. Anteriormente, se han sugerido posibles orígenes del surgimiento de las células troncales neoplásicas y aquí se mencionan las investigaciones recientemente reportadas, dado que, al contar con un mejor entendimiento del comportamiento celular, se va comprendiendo el mecanismo de la respuesta ante el tratamiento. Se comentan también los marcadores proteicos encontrados mayormente en las células troncales neoplásicas y cancerosas, ya que algunos estudios se centran en algún tipo particular de expresión. 

## MÉTODOS

Este trabajo recoge una revisión de la literatura con miras a describir información disponible acerca del carcinoma oral de células escamosas, la presencia de células troncales cancerígenas en estas neoplasias, su asociación con el curso de la enfermedad y sus aplicaciones terapéuticas actuales y nacientes. 

**Fig. 1:**
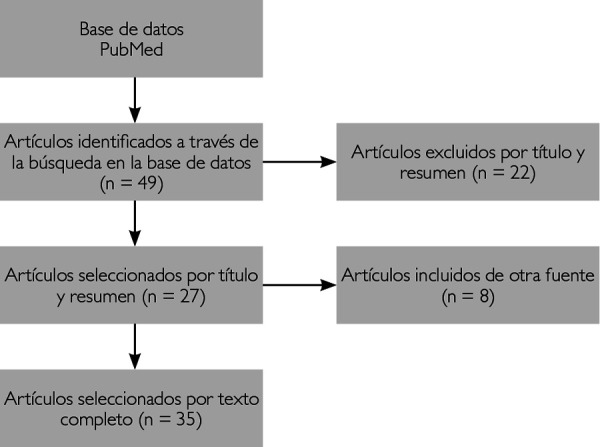
Diagrama de flujo PRISMA para la selección de artículos. Fuente directa.

Se realizó una búsqueda avanzada en la base de datos PubMed introduciendo el siguiente algoritmo de búsqueda, sin filtros adicionales: *(((neoplastic stem cells [ MeSH Terms ]) OR (Cancer stem cells [ Text Word ])) AND (Squamous Cell Carcinoma of Head and Neck [ MeSH Terms ])) AND (Oral squamous cell carcinoma [ Text Word ])*. Se obtuvo un resultado de 49 artículos publicados entre los años 2012 y 2022. De estos, se eligieron 27 según el título y el resumen en su asociación con el tema. Se incluyeron 8 artículos adicionales sugeridos por su relación con la información previamente buscada, con lo que se obtuvo un total de 35 artículos (Fig. 1).

## RESULTADOS

De acuerdo con la estrategia de búsqueda empleada se incluyeron veintisiete artículos y, adicionalmente, se añadieron a la revisión ocho artículos más, sugeridos por su relación con la información previamente buscada. El total de los artículos fue revisado y evaluado por los autores para la extracción de la información relevante que se reporta en los párrafos a continuación. 

### Células troncales cancerígenas

El análisis y diagnóstico histológico de las neoplasias muestra que existen características histológicas importantes, entre las cuales se encuentra la diferenciación de las células que componen el tumor, y que es un indicador que predice metástasis y recurrencia tumoral. Las células troncales neoplásicas están implicadas en la carcinogénesis y el comportamiento tumoral. El fenotipo indiferenciado de estas células se correlaciona con las propiedades de tumorigénesis, invasión, recaída y resistencia a los fármacos ^9^^,^^10^.

Existen dos hipótesis principales sobre el origen de las células troncales cancerígenas: la desdiferenciación de las células tumorales o la derivación de las células madre normales mediante cambios genéticos o epigenéticos. El proceso de transición epitelio-mesénquima permite a las células troncales cancerígenas sufrir cambios fenotípicos por los que se vuelven más móviles ^5^^,^^11^.

Se ha asumido que la actividad de los brotes tumorales, pequeños grupos de células tumorales, representa una característica de la transición epitelio-mesénquima. La evidencia sugiere que las células que atraviesan la transición epitelio-mesénquima adquieren capacidades similares a las de las células madre y pueden nombrarse células madre cancerígenas ^12^.

Las células madre o células troncales cancerígenas o neoplásicas tienen la capacidad de autorrenovación, de diferenciarse en líneas celulares tumorales heterogéneas y con características fenotípicas de células troncales. Se agrupan en los anteriormente mencionados brotes tumorales, que se localizan en el frente de invasión tumoral. Esto manifiesta características del comportamiento agresivo de la neoplasia, como la pérdida de adhesión celular y la invasión local ^13^.

### Transición epitelio-mesénquima

**Fig. 2:**
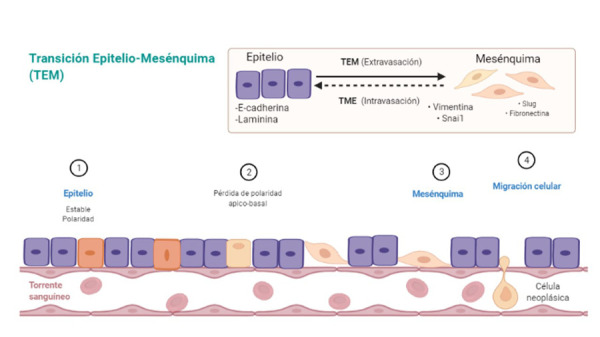
La reorganización de la actina del citoesqueleto es fundamental para la transdiferenciación de las células epiteliales en células mesenquimales móviles (transición epitelio-mesénquima). Las células pierden contacto con la membrana basal durante la invasión y la intravasación en los vasos sanguíneos y linfáticos, y la extravasación en sitios distantes ^15^ (Fuente directa).

Una característica histológica considerada esencial antes de que ocurra la invasión tumoral y la metástasis es la transición epitelio-mesénquima (TEM), que puede describirse como el cambio de las propiedades de las células epiteliales del carcinoma a células mesenquimales. Esto hace que se pierda la adhesión célula a célula y se desarrolle un fenotipo migratorio en dicha transición ^14^ (Fig. 2).

**Fig. 3:**
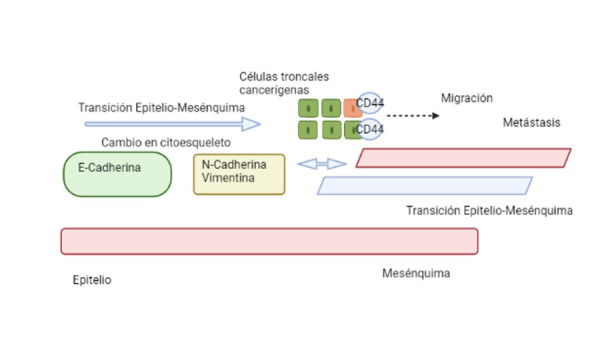
Las poblaciones de células troncales cancerígenas presentan fenotipos de transición epitelio-mesénquima. Se muestra la expresión de marcadores de células troncales en carcinomas orales de células escamosas, con influencia en su comportamiento (Fuente directa).

Se ha reportado información que apoya una relación existente entre las células troncales cancerígenas y la transición epitelio-mesénquima, ya que las células troncales cancerígenas manifiestan fenotipos de transición epitelio-mesénquima, y esta última es importante para la presencia de características similares a las de las células troncales ^6^. Esta reciprocidad pone de manifiesto una relación patológica de retroalimentación positiva, que ha podido asociarse con la resistencia a la terapia, ya sea quimioterapia o radioterapia, así como la progresión de la enfermedad, la recurrencia y, como resultado, un mal pronóstico general ^16^ (Fig. 3).

Como se ha mencionado, las células cancerosas pierden su adhesión celular y son capaces de atravesar la membrana basal, con lo que llegan a entrar al torrente sanguíneo a través de la extravasación. Posteriormente, estas células pueden formar micrometástasis, donde experimentan una transición mesénquima-epitelio para el crecimiento clonal. Por tanto, estas transiciones representan su involucramiento en la cascada de la invasión y la metástasis.

En su estudio, Xu *et al*. ^17^ evalúan el efecto del factor de crecimiento epidérmico (EGF) sobre las células COCE (células de carcionoma escamoso oral), en las que estas sufrieron cambios morfológicos de tipo mesenquimal, característicos del fenotipo de la TEM. Esto se confirmó, además, por una disminución dependiente de la dosis de la expresión de E-cadherina, un marcador de superficie celular específico de las células epiteliales, junto con un aumento simultáneo de la expresión de vimentina, un marcador relacionado con el mesénquima. Se ha reportado que más del 90% de los carcinomas de células escamosas de cabeza y cuello sobreexpresan los receptores del factor de crecimiento epidérmico (EGFR), que desempeñan un papel importante en la progresión del tumor y la resistencia al tratamiento.

### Marcadores de inmunohistoquímica

La carcinogénesis involucra procesos complejos, muchos de los cuales permanecen sin ser esclarecidos. En este sentido, un componente que ha resultado relevante es el papel de las células troncales cancerígenas en el carcinoma oral de células escamosas. Se sostiene que agrupaciones pequeñas de células troncales cancerígenas se encuentran entre la población de las células neoplásicas que componen el tumor. El funcionamiento molecular de estas células continúa en estudio. Se ha demostrado la expresión de marcadores de células troncales en carcinomas orales de células escamosas, tales como OCT4, NANOG y CD-33. Por otra parte, también se identificó la reactivación de la vía Wnt/β-catenina en el cáncer oral. Esta vía es una de las principales involucradas en la regulación de la proliferación y la diferenciación de las células troncales ^18^^,^^19^.

NANOG es un factor de transcripción con homeodominios y que está involucrado en el mantenimiento de la pluripotencia y la autorrenovación en células madre embrionarias. Sin embargo, su influencia en el carcinoma oral de células escamosas no se conoce del todo. Se ha sugerido que puede ser un predictor frente al riesgo del desarrollo de una neoplasia maligna. En su estudio, Kashyap *et al*. reportaron la expresión de NANOG en aquellos carcinomas de células escamosas cuyos ganglios linfáticos mostraban positividad y se había registrado resistencia a los fármacos. Los resultados apoyan a NANOG como un biomarcador en estadios avanzados, ya que la expresión fue baja en tejidos adyacentes al tumor o en estadios tempranos ^20^.

Además, se han utilizado otros marcadores que aportan a la identificación de células madre cancerígenas, como la glicoproteína de transmembrana CD44, receptora de ácido hialurónico y que se ha implicado en la adhesión, migración y metástasis de las células cancerosas ^21^^,^^22^. Sagharavanian *et al*., en su estudio, demostraron que la sobreexpresión de la molécula de adhesión CD44 manifestaba una asociación significativa con carcinoma de células escamosas de mayor grado ^23^.

Asimismo, se ha identificado la expresión de aldehído deshidrogenasa-1 (ALDH1), enzima con localización en citosol, que tiene como función promover la oxidación de los aldehídos dentro de la célula y ayuda a la oxidación del retinol a ácido retinoico en las etapas iniciales de la diferenciación de las células troncales. El ácido retinoico se encuentra involucrado en la gestión de la proliferación celular. También los subproductos del proceso metabólico aumentan la capacidad de autorrenovación de las células troncales mesenquimales. Es, por tanto, un marcador útil para la identificación de células que recuerdan a células troncales neoplásicas. Además, su expresión ha sido asociada con la resistencia a la terapia del carcinoma de células escamosas, así como con un pronóstico más desfavorable ^24^.

### Curso de la investigación en el entendimiento del comportamiento tumoral y desarrollo de potenciales tratamientos

Se ha propuesto la necesidad del desarrollo y la adopción de nuevas medidas terapéuticas dirigidas a la eliminación de las células troncales cancerígenas, que en muchos casos se han mostrado resistentes al tratamiento por quimio y radioterapia.

Los recientes avances en la biología celular y molecular han hecho posible y cada vez más viable el desarrollo y empleo de medicina personalizada. Como es sabido, las neoplasias malignas tienen lugar ante un muy variable número de alteraciones genéticas y afectaciones múltiples en el control de diversas funciones celulares, además de las variadas asociaciones etiológicas y los factores de riesgo. Se tiene como resultado un componente tumoral celular y molecular en demasía heterogéneo.

A partir de esta premisa, varios son los flancos desde donde se idean potenciales tratamientos que muestran resultados favorables. Dentro de este conjunto, las modificaciones epigenéticas y las alteraciones postraduccionales de las proteínas se han convertido en nuevos objetivos de las terapias contra el cáncer, como lo reportan Marques *et al*. en su trabajo de evaluación de Entinostat ^25^.

El entinostat es un inhibidor altamente selectivo de la histona desacetilasa 1 (HDAC1) y HDAC3. La eliminación de los grupos acetilo de las colas de las histonas da lugar a una unión más fuerte entre el ADN y el núcleo de las histonas nucleosómicas, lo que limita el acceso de los reguladores transcripcionales a los genes diana. Por lo tanto, las histonas no acetiladas suelen estar asociadas a la inactividad transcripcional. La expresión de los genes suprimidos puede restablecerse mediante la inhibición de la actividad de la histona desacetilasa ^26^.

La acetilación del grupo ɛ-amino presente en los residuos de lisina de la histona desempeña un papel fundamental en la transcripción de los genes y la supervivencia celular. Las histonas acetiltransferasas y las histonas desacetilasas controlan los niveles de acetilación de las histonas y la accesibilidad de la cromatina ^27^.

Anteriormente, se ha evaluado el efecto de entinostat sobre las células troncales cancerígenas con el uso de células de carcinoma oral de células escamosas, bien caracterizadas con marcadores CD44 y ALDH. Se observó una reducción de más de la mitad de las células troncales cancerígenas después de la administración, así como una reducción en la proliferación de células neoplásicas, seguida por arresto del ciclo celular en fase G0/G1, y una notable apoptosis en células tumorales. Asimismo, se identificó un incremento en la acetilación de histonas H3 y H4 ^25^.

Por otra parte, el Atlas del Genoma del Cáncer publicó el análisis del genoma del carcinoma de células escamosas de cabeza y cuello, en el cual se pone de manifiesto la existencia de mutaciones en el DNA como alteraciones en el número de copias de genes y alteraciones en los genes principalmente expresados, deleciones y translocaciones ^28^.

En un estudio en el que se analizó un panel de 42 genes que codifican para las principales proteínas susceptibles de ser tratadas con fármacos en una muestra de pacientes con carcinoma de células escamosas de cabeza y cuello, se encontraron 6 genes blanco relevantes, que incluyen PGF, PDL1/CD274, CDK6, EGFR, MET y VEGFA. Estos genes están involucrados en vías tumorigénicas cruciales y, por lo tanto, sus proteínas asociadas podrían representar potenciales blancos para terapias farmacológicas dirigidas ^29^.

Desde hace varios años, se identificó que la mayoría de los carcinomas de células escamosas de cabeza y cuello expresan en la superficie de las células tumorales un alto grado EGFR, por lo que se siguen analizando opciones de terapia ^30^.

Muchos tumores resistentes a la terapia se originan en células tumorales que tienen un fenotipo de transición epitelio-mesenquimal. Las células epiteliales pierden su polaridad y adquieren un fenotipo mesenquimal migratorio. EGFR suele estar sobreexpresado y ha sido considerado como factor de control en el fenotipo maligno en los carcinomas de células escamosas de cabeza y cuello, mediante el ajuste de las moléculas implicadas en los procesos invasivos. 

Sin embargo, un estudio reportó que la capacidad de invasión estaba inversamente correlacionada con la expresión del EGFR en pacientes con carcinoma oral de células escamosas. Los resultados sugieren que la pérdida de expresión del EGFR se asocia con la adquisición de un fenotipo invasivo. No obstante, se sigue requiriendo de más investigaciones al respecto ^31^.

Desde hace algunos años, los marcadores de superficie se han estudiado como posibles objetivos farmacéuticos de las células troncales cancerígenas. La progresión de esta neoplasia está asociada con las células troncales cancerígenas Bmi1-positivas, responsables de la invasión tumoral, la resistencia a los fármacos y la metástasis en los ganglios linfáticos. Por ende, Bmi1 es un posible blanco terapéutico para las células troncales cancerígenas de este carcinoma. Se han reportado enfoques de la inmunoterapia, como vacunas, infusión de células T, inhibidores de puntos de control y anticuerpos monoclonales específicos ^32^.

### Perspectivas a futuro

Recientemente, se ha investigado sobre otra modalidad de tratamiento en el combate contra el cáncer, la hipertermia local, que ha evolucionado a la aplicación de nanopartículas magnéticas como fuente de calor y es llamada hipertermia magnética. Se ha estudiado el empleo de nanopartículas de óxido de hierro supermagnético, cuyo depósito se realiza directamente dentro del tumor y que actúa como transductor para convertir la energía electromagnética en calor, al ser expuesto a un campo magnético alterno ^33^. La hipertermia con fluido magnético utiliza nanopartículas magnéticas bajo un campo magnético alterno para generar calor mediante la rotación del vector magnético y la rotación física. Este fluido puede ser administrado a través de una arteria de alimentación del tumor o por inyección directa. Una vez que ha sido interiorizada por las células mediante endocitosis, bajo el campo magnético alterno externo se forma rápidamente una zona de alta temperatura dentro de las células tumorales, que logra la muerte de estas o la inducción de la apoptosis. Una ventaja es que se evita que los tejidos normales que rodean al tumor sean afectados por el calor ^34^.

Se ha estudiado la posibilidad de una terapia dirigida a células madre cancerígenas que sobreexpresan CD44 con nanopartículas superparamagnéticas de óxido de hierro. Se observó que estas fueron eficientemente internalizadas por las células troncales cancerígenas y que indujeron la muerte programada de las células al ser sometidas al campo magnético alterno. Algunos estudios han confirmado que las células tumorales son más sensibles al calor que las células normales, sobre todo en el centro de la neoplasia, que muestra un estado de baja nutrición e hipoxia ^35^.

Las terapias dirigidas a las células troncales cancerígenas son consideradas eficaces para el tratamiento del cáncer, aunque aún se tienen como métodos muy jóvenes que requieren ensayos y estudios que los coloquen como tratamientos seguros y con mayor aplicabilidad.

## DISCUSIÓN

El carcinoma de células escamosas de cabeza y cuello incluye tumores malignos de aparición en la cavidad oral, orofaringe, nasofaringe, cavidad nasal, laringe y glándulas salivales. En tales neoplasias, se han identificado estas agrupaciones de células descritas con un comportamiento biológico agresivo, con capacidad de autorrenovación y con la facultad de generar una población heterogénea de células cancerosas. Este tipo de células manifiestan características semejantes a las de las células troncales, por lo que se las ha denominado células troncales cancerígenas, las cuales se han relacionado como una de las causas de recurrencia y metástasis. Como se ha comentado, las células troncales cancerígenas comparten características con las células troncales normales, entre ellas el estado indiferenciado, y se ha sugerido que las células troncales cancerígenas o neoplásicas surgen a partir de células cancerosas no troncales mediante un mecanismo de reprogramación.

Muchos de los factores de transcripción clave, tales como OCT4 y SOX2, que median en el mantenimiento del fenotipo de células troncales en las células troncales embrionarias y somáticas, también se sobreexpresan en las células troncales cancerígenas.

Para el tratamiento de este padecimiento, se emplean terapias multimodales, principalmente cirugía, además de quimioterapia y radioterapia. No obstante, se reporta que, en muchos casos, para la enfermedad localmente avanzada, la quimioterapia tiene una tasa de éxito pobre. Asimismo, los pacientes con enfermedad recurrente o con metástasis tienen un pronóstico desfavorable, y pocas opciones de tratamiento eficaces.

Se han propuesto y desarrollado terapias dirigidas a la eliminación de las células troncales cancerígenas. Los marcadores de superficie celular se han estudiado como objetivos farmacéuticos de las células troncales cancerígenas. La localización de células altamente tumorigénicas podría contribuir al desarrollo de nuevas estrategias terapéuticas y, por ende, ayudar a la disminución de la morbilidad y la mortalidad asociadas con el padecimiento en cuestión.

## CONCLUSIONES

Los pacientes con carcinoma oral de células escamosas, con frecuencia, sufren recurrencia de la enfermedad a nivel local o a distancia, con falla en el tratamiento con quimio y radioterapia. Este comportamiento desfavorable se ha asociado con la presencia de células troncales neoplásicas, que se han identificado como pequeñas subpoblaciones de células tumorales indiferenciadas, las cuales se involucran en el inicio y la progresión de la neoplasia maligna, y que además sobreviven tras la resección quirúrgica y la terapia. Durante la evolución del tumor, las células troncales neoplásicas adquieren características que les hacen transcurrir a través de la transición epitelio-mesénquima, por lo que pueden migrar hacia tejidos adyacentes e invadir vasos sanguíneos y linfáticos. Se ha demostrado la expresión de marcadores de células troncales en carcinomas orales de células escamosas, los mismos que han aportado a la identificación de estas y se han implicado en el comportamiento de las células cancerosas. Se han propuesto y desarrollado nuevas medidas terapéuticas dirigidas a la eliminación de las células troncales cancerígenas.

## Financiamiento:

La presente investigación no ha recibido ninguna beca específica de agencias de los sectores públicos, comercial o sin ánimo de lucro
